# Mr. Ring, on the Cow-Pox

**Published:** 1803-12-01

**Authors:** John Ring

**Affiliations:** New Street, Hanover Square


*To the Editors of the Medical and Physical Journal.*

Gentlemen,

In the foregoing account, we are not informed at what period the matter was taken, nor whether it was inserted in a fluid state; and consequently, notwithstanding I entertain a high opinion of Mr. Weston's abilities, it is impossible to determine whether the failure in the hot season was inevitable.

I cannot dissemble my own opinion, which is, that if the virus be taken as soon as it is secreted, and immediately inserted, vaccination may be perpetuated in any part of the globe.

But it is necessary to bear in mind, that in Negroes, the progress of the disease is more rapid than in Whites. In the former, the desiccation of the pustule takes place on the sixth day. Such, at least, was the result of experiments made in Paris; of which I have given the particulars in my Treatise on the Cow-pox, p. 791.

From this statement it appears, that in Negroes, the vaccine fluid should not in general be taken later than the fourth or fifth day.

In the beginning of April, when I was inoculating at the Central House of the Royal Jennerian Society, previous to the appointment of a resident Inoculator, I supplied Mr. Bulmer of the Strand with some cow-pock matter on vaccinators, for Dr. Longmore of Quebec; which he inserted in the month of June with success.

In April, 1802, Dr. Longmore had introduced vaccination into Quebec, by means of matter received from a gentleman of Richmond, whose name is not mentioned. This matter was ninety-one days old.

From that stock he inoculated nearly fifty persons; and other practitioners, to whom he gave the virus, inoculated with equal success. In consequence of the heat of the summer, and the want of a succession of patients, the virus lost its virtue; and Dr. Longmore informs us, in an Address to the Public on Vaccination, published in the Quebec Gazette, for the 11th of August last, that although, since the period alluded to, he had made repeated attempts to procure a fresh supply from England, he could never again inoculate with effect with any matter he received, till the arrival of that which produced the affection in June last.

When Dr. Longmore published his Address he had vaccinated upwards of thirty patients with the virus he received from me; and with the same complete success as in the preceding year. He had also put the patients vaccinated, in both seasons, to the most severe tests of variolous infection, both in the natural and artificial way.

In his Address he offers to inoculate the inhabitants of Quebec gratis, twice a week, at the Hotel Dieu in that city.

The introduction of the new practice into Canada is of the more importance, on account of the severity of the small-pox in that country; for Dr. Longmore informs me, that when it prevails there, even in those cases where, from the humanity of the British government, every indulgence both with regard to medical assistance and other necessaries, is extended to those who labour under the disease, it generally proves confluent and fatal. This is supposed to proceed from their habits of life, and from the paint and other substances with which their skins are besmeared.

From the enquiries which Dr. Longmore has hitherto made, it does not appear that the cow-pox in the cow is known in Canada.

One

One practical observation which he has made, both in the inoculation of the small-pox, and in that of the cow-pock, is, that those persons who have the fairest skin are the most susceptible of infection.

I lately received from Dr. De Carro, a work entitled The History of Vaccination in Turkey, Greece, and the East Indies. This publication has no tendency to corroborate the opinion, that the cow-pock is a preventive of the plague. It rather confirms the opinion I gave in my Treatise, p. 1026

An instance is there given of a child vaccinated by Dr. La Font, a French physician of Salonica, who succeeded in producing the cow-pock by inoculating a cow with equine matter, as others had done before, in England and in Italy. Two other patients of Dr. La Font have since had the plague; and one of them died of the disease.

By a letter which I have just received from Dr. Jenner, I find, that he has little or no confidence in the statement lately given of the anti-pestilential virtue of the cow-pock. It is, indeed, an opinion not warranted by analogy, nor by sufficient evidence.

I have reason to believe, that in the accounts lately transmitted, there is great exaggeration; and it is much to be regretted, that men of learning and ingenuity should hazard the lives of themselves and others, in such vain experiments, and visionary pursuits.

*New Street, Hanover Square,* I am, &c.

*November* 19, 1803. JOHN RING.

